# *S*-Methyl-(2-Methoxycarbonylamino-Benzimidazole-5) Thiosulfonate as a Potential Antiparasitic Agent—Its Action on the Development of *Ascaris suum* Eggs In Vitro

**DOI:** 10.3390/ph13110332

**Published:** 2020-10-23

**Authors:** Małgorzata Dmitryjuk, Magdalena Szczotko, Katarzyna Kubiak, Radosław Trojanowicz, Zhanna Parashchyn, Halyna Khomitska, Vira Lubenets

**Affiliations:** 1 Department of Biochemistry, Faculty of Biology and Biotechnology, University of Warmia and Mazury in Olsztyn, Oczapowskiego 1A, 10-719 Olsztyn, Poland; magdalena.szczotko@uwm.edu.pl (M.S.); radoslaw.trojanowicz@student.uwm.edu.pl (R.T.); 2 Department of Medical Biology, Collegium Medicum, School of Public Health, University of Warmia and Mazury in Olsztyn, Zolnierska 14c, 10-561 Olsztyn, Poland; katarzyna.kubiak@uwm.edu.pl; 3 Department of Technology of Biological Active Substances, Pharmacy and Biotechnology, Lviv Polytechnic National University, Bandera 12, 79013 Lviv, Ukraine; zhanna.d.parashchyn@lpnu.ua (Z.P.); halyna.m.khomitska@lpnu.ua (H.K.); vlubenets@gmail.com (V.L.)

**Keywords:** *Ascaris suum*, *S*-methyl-(2-methoxycarbonylamino-benzoimidasole-5) thiosulfonate, benzimidazole, drug resistance, ovicide

## Abstract

*Ascaris suum* is a soil-transmitted parasite causing ascariasis in pigs, largely limiting livestock production globally. Searching for new drugs affecting all stages of nematode development is necessary and widely postulated. The in vitro activity of *S*-methyl-(2-methoxycarbonylamino-benzoimidasole-5) thiosulfonate on *A. suum* developing eggs was studied. Five concentrations of the drug were used—0.625, 1.25, 2.5, 5 and 10 mM during 24, 48 and 72 h of exposure. After drug treatment, the eggs were washed and cultured in 0.05 M HCl at 27 °C for 20 days. Both the concentration and duration of the drug exposure had an inhibitory impact on the percentage of L_2_ larvae developed. The best effect was obtained after 72 h of incubation in 5 mM drug solution, only 1.9 ± 3.3% of the larvae developed to the L_2_ stage. Moreover, no SNP was detected at codon 167, which is correlated with benzimidazole resistance, in the tested samples. For the first time, it has been demonstrated that *S*-M-(2-MKA-BZ-5)TS seems to be a potential ovicidal anti-helminthic agent. It may lead to the elimination of parasites and reduce environmental contamination from roundworm eggs. The ovicidal effects of the drug should be additionally confirmed by further infection studies using experimental animals.

## 1. Introduction

*Ascaris suum* Goeze, 1782, is the most widespread intestinal parasite causing ascariasis in pigs. This soil-borne infection has a high worldwide prevalence and causes significant economic losses. It manifests by reducing the swine production efficiency and condemnation to organs such as the liver [1]. *A. suum* is often postulated as a model for research on human infection by *Ascaris lumbricoides* Linnaeus, 1758. Human ascariasis affects approximately 820 million people, most often the younger generations, especially in areas with poor access to water, sanitation and hygiene [2,3,4,5]. It is standard to use large-scale preventive chemotherapy to control for geohelminths due to the large epidemic threat [6]. Benzimidazoles (BZ), imidazothiazoles/tetrahydropyrimidines (LV) and the macrocyclic lactones (ML) are three main classes of synthetic chemicals used to control gastrointestinal helminth infections [7,8]. BZ is a group of drugs most commonly used to treat soil-transmitted worms. Their widespread use increases the possibility of developing resistance to BZ. In several helminths, a single nucleotide polymorphism (SNP) in the β-tubulin isotype 1 gene at codon position 200 (TTC/Phenylalanine → TAC/Tyrosine), 167 (TTC, TTT/Phenylalanine → TAC, TAT/Tyrosine) or 198 (GAG, GAA/Glutamic acid → GCG, GCA/Alanine) has been known to cause BZ resistance. However, mutations at codon 198 and 200 have never been described for *Ascaris*. Instead, a mutation at codon 167 has been detected quite often in roundworms [6,9,10].

The activity of anthelmintic drugs is generally directed against larval and adult stages of helminths. However, anthelmintics may also express ovicidal activity, as has been reported in benzimidazoles [11]. The eggs represent the dispersion stage during the life cycle of *A. suum* and are the most resistant form in the development of the nematode [12]. The embryonic development pattern in roundworm eggs has been characterized for nematodes as: cleavage is total and unequal, during gastrulation blastomeres collapse inward, the embryos elongate and form the larvae L_1_ which becomes the L_2_ larvae. The larva then passes through the second molt within the egg and enters into the L_3_ invasive stage. From the environment, invasive eggs per os get into the digestive system of the host, where they are released from the casings and start multi-system wandering to finally settle as adults in the intestine [13,14,15].

Searching for new drugs affecting all stages of nematode development is advisable. Currently, a search for new biologically active substances effective against worms, including those of natural origin, has often been sought [16,17,18]. It should be noted that allicin (diallyl thiosulfinate) is characterized by such properties. Thiosulfinates RS(O)SR are volatile sulfur-containing compounds found in garlic and other *Allium* species. These compounds are unstable during storage [19]. Structural analogs of allicin are thiosulfonates RS(O)_2_SR’, which are stable biologically active compounds with a broad spectrum of action [20,21]. Synthetic esters of thiosuloacids (ETS) are highly reactive compounds that interact mainly by nucleophilic substitution reactions occurring with the breaking of an S-S-bond due to the redistribution of electron density in the thiosulfone group that determines the direction of nucleophile attack. Therefore, ETS affects the metabolism of nucleic acids, which contain amino groups, as well as proteins and amino acids with disulfide and sulfide groups [22]. Thiosulfonates also have a positive influence on metabolic processes in different tissues of rat organisms [23,24]. Furthermore, a thiosulfonate derivative of benzimidazole was tested for its effectiveness as an anticancer agent and showed significant growth inhibition against human tumor cell lines derived from several neoplastic diseases, e.g., leukemia, breast or prostate cancer [25].

In view of the above, the use of the sulfur derivative of benzimidazole to control the most resistant developmental stage of *A. suum*—its eggs—seems to be promising. Especially due to the fact of increasing drug resistance among nematodes against known anthelmintics. Therefore, the aim of the present study was to demonstrate the in vitro antiparasitic activity of *S*-methyl-(2-methoxycarbonylamino-benzoimidasole-5) thiosulfonate (*S*-M-(2-MKA-BZ-5)TS) using *A. suum* developing eggs. Taking into account the occurrence of the genetically determined BZ resistance in nematodes, we additionally checked whether the eggs isolated from the roundworms studied showed drug resistance to benzimidazole associated with the mutation at codon 167 in the β-tubulin gene.

## 2. Results

### 2.1. Egg Development after Drug Treatment

**Figure 1:**
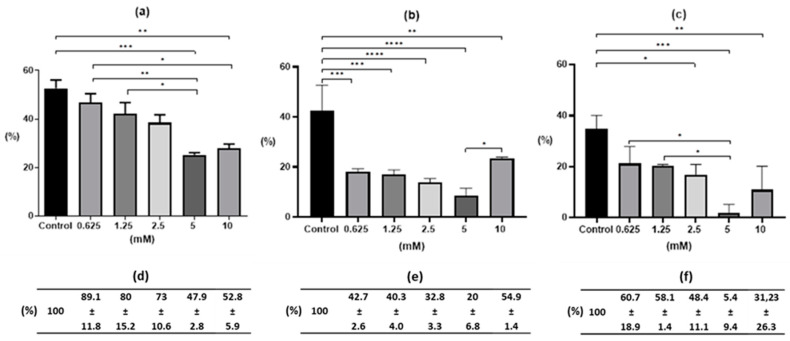
Percentage of L_2_ larvae from *Ascaris suum* egg in vitro incubation according to time exposure and *S*-M-(-2MKA-BZ-5)TS concentration: (**a**) 24h-; (**b**) 48h-; (**c**) 72h-drug exposure. Notes: * significant difference at *p* < 0.05; ** significant difference at *p* < 0.01; *** significant difference at *p* < 0.001; **** significant difference at *p* < 0.0001. (**d**,**e**,**f**)—percentage of L_2_ larvae relative to the Control (0.1% DMSO) expressed as 100% after 24, 48 and 72 h exposure, respectively.

Both the concentration of the S-M-(-2MKA-BZ-5)TS and the time of exposure to the drug had an impact on the percentage of invasive larvae developed. After 24-h of drug exposure and 20-days of egg cultivation, there was a statistically significant decrease in the number of invasive larvae at concentrations of 5 and 10 mM of the study drug, as compared to the control (25.2 ± 1.5%, *p* < 0.001, and 27.8 ± 3.1%; *p* < 0.01, respectively, Figure 1a). After 24-h of drug treatment, statistically significant differences were also noted between the data obtained for 0.625 mM and 5 mM (*p* < 0.01) and 10 mM (*p* < 0.05), and between 1.25 mM and 5 mM (*p* < 0.05) drug concentration (Figure 1a). At the highest drug concentrations (5–10 mM), the effect was optimal in comparison to the control (expressed as 100%). Both concentrations were comparable and amounted to approximately half of the value recorded for the control sample (Figure 1d).

After 48 h of drug action, the greatest effect was obtained at lower drug concentrations, namely in 0.625, 1.25 and 2.5 mM solutions. There were statistically significant differences in relation to the control (*p* < 0.001, *p* < 0.001, *p* < 0.0001, respectively; Figure 1b) and the number of developed larvae was lower (18.1 ± 1.1%, 17.1 ± 1.7%, 13.9 ± 1.4%, respectively; Figure 1b) than after 72 h of drug treatment (21.2 ± 6.6%, 20.3 ± 0.5%, 16.9 ± 3.9%; Figure 1c), where statistically significant differences (*p* < 0.05) were noted only at 2.5 mM drug concentration, as compared to the control. After two days of drug action, a significant decrease in the percentage of invasive larvae in the medium of 5 mM (8.5 ± 2.9%) and a moderate decrease at 10 mM drug concentration (23.3 ± 0.6%) was noted. In both cases of the highest drug concentrations, statistically significant differences were noted (*p* < 0.0001, *p* < 0.01, respectively). After 48 h, there was a further decrease in the number of motile larvae in eggs at concentrations of 0.625 to 5 mM, when compared to the control and expressed as 100%. In the 10 mM drug solution, the percentage of L_2_ larvae remained at the level obtained after 24 h of treatment (Figure 1e).

After 72 h of incubation in 5 mM drug solution, the smallest percentage of invasive larvae was observed within the entire experiment (1.9 ± 3.3%, *p* < 0.001 vs. Control), while the 10 mM drug solution did not give as strong of a larval inhibitory effect (10.9 ± 9.2%, *p* < 0.01 vs. Control; Figure 1c). On the third day of drug treatment, the effectiveness of the drug decreased at the lowest three concentrations relative to the control expressed as 100%. In replicates of the 10 mM trials, quite mixed results were recorded, which resulted in a large standard deviation (31.23 + 26.3%). The greatest effect was observed at the 5 mM concentration, as compared to the control (Figure 1f).

**Figure 2:**
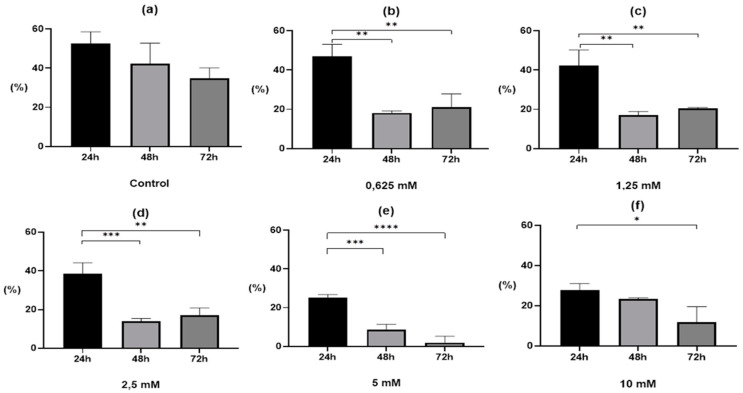
Percentage of invasive larvae (L_2_) depending on the time of S-M-(-2MKA-BZ-5)TS exposure at individual drug concentrations: (**a**) Control—0.1% DMSO; (**b**) 0.625 mM; (**c**) 1.25 mM; (**d**) 2.5 mM; (**e**) 5 mM; (f) 10 mM. Notes: * significant difference at *p* < 0.05; ** significant difference at *p* < 0.01; *** significant difference at *p* < 0.001; **** significant difference at *p* < 0.0001.

**Figure 3:**
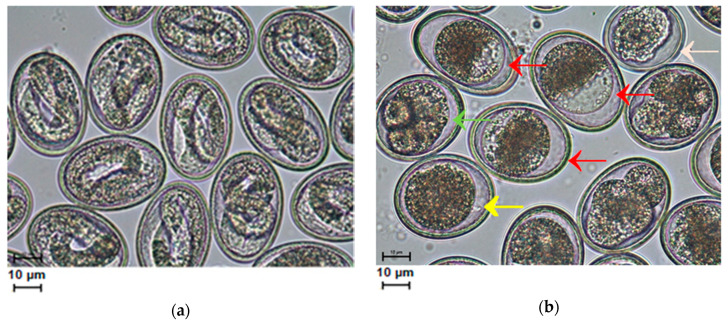
Developmental degree of *Ascaris suum* eggs after 72 h exposure to the drug and 20 days of in vitro culture in 0.05 M HCl: (**a**) Control test, 0.1% DMSO—eggs mainly in the stage of correctly developed larvae L_2_; (**b**) 10 mM solution of the drug—egg development was largely arrested at the one-cell stage (**←**) or irregular cell division (**←**) was observed, it resulted in atypical blastomeres inside (**←**) and in a change in the shape of the eggs (**←**) (40× magnification, scale 10 μm).

The 0.1% DMSO solution had a slightly inhibitory effect on larvae development. In control samples, a gradual decrease in the number of invasive larvae was observed during the experiment, but these were not statistically significant differences. After 24 h, 52.6 ± 5.9% L_2_ larvae were recorded in the control sample, after 48 h 42.4 ± 10.3%, and after 72 h 34.9 ± 5.2% (Figure 2a). However, DMSO did not affect the correctness of embryogenesis (Figure 3a). Lower drug concentrations (0.625–2.5 mM) achieved a better inhibitory effect on the development of larvae after 48 h of treatment than after 72 h, although in both cases there were statistically significant differences (Figure 2b–d). At the two highest concentrations, the percentage of L_2_ larvae gradually decreased with the length of incubation. A much clearer effect was observed for the 5 mM drug concentration, where statistically significant differences were observed in the ratio 48 h and 72 h vs. 24 h (*p* < 0.001 and *p* < 0.0001, respectively; Figure 2e) than in the 10 mM solution, where statistically significant differences were only observed between 72 h and 24 h of the experiment (*p* < 0.05; Figure 2f).

After the longest exposure to *S*-M-(-2MKA-BZ-5)TS and the highest drug concentrations (5, 10 mM), in addition to the inhibition of egg development, blastomere distortion, embryo degradation and egg deformation were observed (Figure 3b); whereas the L_2_ larvae were properly formed in the control sample (Figure 3a).

### 2.2. β-Tubulin SNP Genotyping

**Figure 4:**
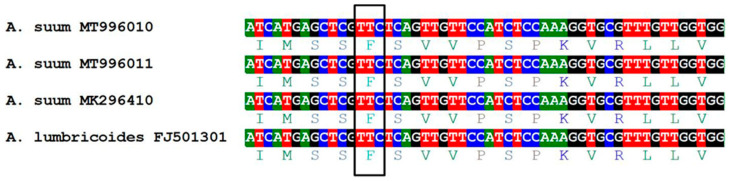
The partial nucleotide and amino acid sequences from the fragment of β-tubulin gene around codon 167 of the selected isolates of *Ascaris suum* eggs obtained in this study (GenBank, MT996010, MT996011) and deposited earlier (GenBank, MK296410, FJ501301). The frame marks codon 167.

In a total of 54 DNA isolates from *A. suum* eggs, a fragment of β-tubulin gene (608 bp), including codon position 167, was successfully amplified. Analysis of the nucleotide and amino acid sequences of two randomly selected PCR positive samples confirmed a high similarity to β-tubulin of *A. lumbricoides* (GenBank, EU814697, FJ501301) and *A. suum* (GenBank, MK296410) (Figure 4).

**Figure 5:**
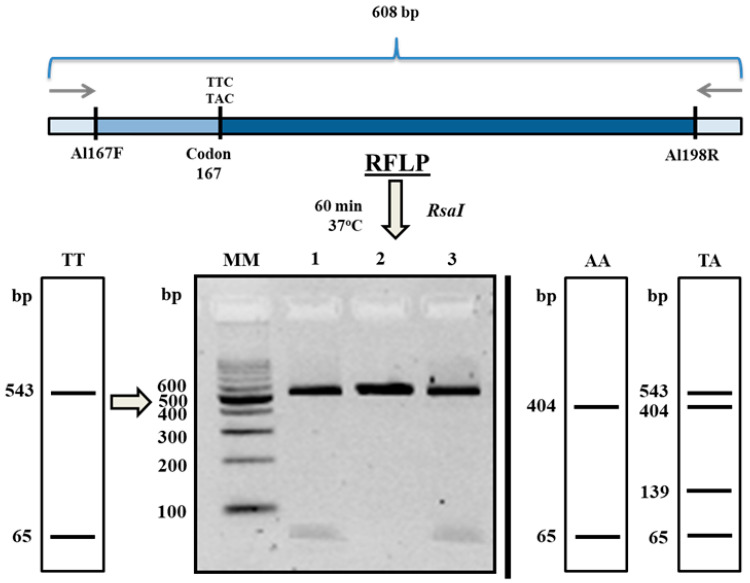
Schematic representation of the PCR-restriction fragment length polymorphism (RFLP) methodology for the screening of β-tubulin polymorphisms of codon 167 in *Ascaris suum* eggs: TT—unmutated homozygous (TTC/TTC); AA—mutated homozygous (TAC/TAC); TA—heterozygous (TTC/TAC), according to Zuccherato et al. [6] after own modification. Notes: MM—molecular markers; 1,3—exemplary *RsaI* restriction cleavage products (548 and 65 bp; TT homozygotes); 2—product before restriction analysis (608 bp).

Polymerase chain reaction-Restriction Fragment Length Polymorphism (PCR-RFLP) analysis in all of the studied pulls of *A. suum* eggs revealed the absence of the 167 codon polymorphism. The restriction patterns obtained after digestion were specific for unmutated, BZ sensitive, homozygotes (TTC/TTC) (Figure 5). Additionally, nucleotide sequences analysis—MT996010, MT996011 (Figure 4), confirmed the absence of the mutation at this position. Due to the lack of polymorphism at codon 167 in tested pulls of 1000 eggs, genotyping of the β-tubulin gene sequences from single eggs was not performed.

## 3. Discussion

Existing anti-parasitic worm drugs have significant limitations such as a limited spectrum of activity across worm species and increasing drug resistance. Due to this, new anthelmintic therapies, whether from newly discovered drugs, improved versions or combinations of existing drugs, are urgently needed [26]. Additionally, the available anthelmintic drugs are mostly not ovicidal, but treatment with their use eliminates worms from infected hosts and thus is contributing to environmental contamination. In other words, such a treatment favors a temporary cure and then reinfection due to the excretion of parasite eggs into the ground through the feces [11,27].

The activity of anthelmintic drugs has been shown against a few different nematode eggs, but effects against *A. suum* eggs have been reported only after in vivo treatment. Boes et al. [11] investigated the in vivo effect of four different anthelmintic drugs (albendazole—ALB, pyrantel pamoate—PYP, ivermectin—IVM and piperazine dihydrochloride—PHC) on subsequent embryonation and infectivity of *A. suum* eggs. These studies showed that egg development appeared normal in cultures from worms of the piperazine, pyrantel and ivermectin treated groups. However, in the albendazole cultures, egg development was largely arrested. The in vitro effect of thiabendazole on *A. lumbricoides* eggs was studied by Massara et al. [27]. Thiabendazole, in the concentration of 10 ppm and after 48 h of drug exposure, stopped completely the embryonation of *A. lumbricoides* eggs. According to the authors, the ovicidal effect of thiabendazole can provide new and more efficient prospects for the treatment of ascariasis by preventing the environmental contamination of excreted invasive eggs. In the present study, we were able to demonstrate in vitro that *S*-methyl-(2-methoxycarbonylamino-benzimidazole-5) thiosulfonate, in 5 mM of drug culture after 72 h of exposure, almost completely inhibited the embryonic development of eggs. Under these conditions, on average about 2% of the embryos reached the L_2_ stage. Unexpectedly, a higher drug concentration (10 mM) inhibited larvae development less in *A. suum* eggs exposed for 72 h duration. Under these conditions, approximately 10% of the larvae transformed into the motile larva L_2_ stage. Increasing the dose of the anthelmintic does not always give a better inhibitory effect. Hu et al. [28] used albendazole as an anti-*A. suum* L_4_ drug in vitro and observed no strong response to the increasing dose of the ALB. The response to this drug was similar over four logarithmic doses (the highest dose was 3.8 mM). Moreover, it seems to us that the drug concentration in the first day of dosing is of key importance. Therefore, if the concentration of the drug is initially too low or too high, the effect will not be satisfactory. It can be assumed that this is related to the structure of the egg shells. Perhaps the eggshell layers become drug-tight over time. In turn, too high a concentration of the drug may cause a greater seal of the shell at the beginning of dosing. Therefore, *S*-M-(-2MKA-BZ-5)TS appears to be a promising anti-helminthic agent in the control of ascariasis using intermediate doses of the drug for at least two days. The ovicidal nature of the drug still needs to be confirmed experimentally. Infection of laboratory animals with eggs previously treated with the drug would give an unambiguous answer to the question of whether the eggs are capable of infecting the host. Based on the microscopic observations made, we dare to say that only a small percentage of eggs could be invasive at higher concentrations of the drug. Most of the eggs contained abnormally built embryos unable to further develop. For example, in two replications of the experiment at a concentration of 5 mM, not a single properly developed L_2_ larva was found.

Due to the preliminary nature of our research, the dosage of *S*-M-(-2MKA-BZ-5)TS was higher than that of benzimidazole drugs used by other researchers [27,28]. In addition to inhibiting the development of eggs, the drug we used in the highest doses (5, 10 mM) caused the destruction of roundworm eggs. The eggs were largely deformed, the blastomeres were atypical, and gastrulation was impaired. Similarly, Boes et al. [11] observed that albendazole causes *A. suum* egg development to be largely arrested at the one-cell stage, and if development does occur, irregular cell division leads to the formation of abnormal blastomeres. A total of 7% of eggs cultured after ALB treatment developed into eggs with full-grown larvae. In the present study, the results were better for the 5 mM drug culture, where approximately 2% of the eggs had reached the invasive larva stage and was similar to the 10 mM solution of the drug, where 10% of eggs had reached an infectious stage. In our preliminary studies, the highest concentration of the drug was a lethal dose for male and female adult roundworms, after 72 h of incubation the adults showed no movement (unpublished data).

This is the second successful use of this thiosulphonate derivative from benzimidazole after its potential anticancer activity had been shown. The *S*-M-(-2MKA-BZ-5)TS showed an impressive toxicity effect against leukemia, melanoma, lung, colon, CNS, ovarian, renal, prostate and breast cancer cell line growth in a five concentration assay over a range from 10^−4^ to 10^−8^ M [25]. In this study, we used higher doses of the drug due to the fact that the eggs are at the most resistant stage of development during *A. suum* ontogenesis [12]. This is, inter alia, due to the fact that the eggs have a three layer casing: a glycoprotein outer layer, a chitin middle layer and an ascarosides inner layer which constitutes a serious barrier to the permeation of chemical compounds [29].

Benzimidazoles have found wide application in the treatment of soil-transmitted helminthiasis. Widespread use of these drugs in a target population leads to drug resistance of parasites and produces resistant off-spring. As mentioned in the introduction, this phenomenon has been associated with the occurrence of SNPs in codons 167, 198 and 200 of the beta-tubulin gene of several helminths [6]. Due to the fact that so far only mutations in codon 167 have been found in *Ascaris* [30], we decided to check whether there was a single nucleotide polymorphism at codon 167 in the population of roundworms we studied. By analyzing the eggs isolated from single individuals, we showed that the mutation in this codon did not occur, all of the individuals produced unmutated, BZ sensitive, homozygous (TTC/TTC) offspring. Summing up, the eggs of roundworms tested by us did not show drug resistance to benzimidazoles. Furthermore, the thiosulphonate derivative of benzimidazole, used in this type of experiment for the first time, seems to be a promising potential ovicidal anti-helminthic agent.

## 4. Materials and Methods

### 4.1. Drug Synthesis

The *S*-methyl-(2-methoxycarbonylamino-benzoimidasole-5) thiosulfonate (*S*-M-(-2MKA-BZ-5)TS) was synthesized at the Department of Technology of Biological Active Substances, Pharmacy and Biotechnology, Lviv Polytechnic National University, Ukraine according to the method described by Parashchyn et al. [31]. The drug was dissolved in dimethyl sulfoxide (DMSO) and diluted to a final concentration of 10 mM, 5 mM, 2.5 mM, 1.25 mM and 0.625 mM. The final concentration of DMSO in the medium was 0.1%.

### 4.2. In Vitro Culture and Preparation of Test Samples

Study material consisted of fertilized eggs isolated from the final uterine section of a single *A. suum* female. Living mature roundworms were isolated from the intestines of pigs in a nearby slaughterhouse and transported in worm ARS medium [32]. In the laboratory, parasites were washed in sterile saline and next in ARS medium containing antibiotics (1.2 million units of penicillin and 1 g of nystatin per liter) for 4 h. After washing, roundworms were dissected and fertilized eggs were collected from the distal 2 cm of the uterus. The egg cultures prepared from individual worms were suspended in 5 mL of the drug at the proper concentration and stored in 15 mL Falcon tubes. Controls were suspended with 0.1% DMSO. For each concentration of the drug and appropriate control samples, eggs from separate uteruses were prepared. The experiments were performed in triplicates. From each pull of eggs isolated from a separate uterus used in the experiment, on average 1000 eggs were also frozen at −70 °C for molecular analysis. The eggs were incubated with the *S*-M-(-2MKA-BZ-5)TS for 24, 48 and 72 h at 27 °C. After 24, 48 and 72 h, the tubes were centrifuged at 200 g for 5 min, washed three times with distilled water and cultured in 5 mL of 0.05 M HCl at 27 °C for 20 days. Egg development was monitored every other day under a NIKON ECLIPSE E200 LED microscope equipped with a NIKON DSFi1 camera (NIKON Corporation, Tokyo, Japan). At the end of the experiment, the number of developed L_2_ larvae in 1000 eggs of each sample was calculated.

### 4.3. DNA Extraction

A total 54 samples from an average of 1000 eggs were moved separately to 2 mL tubes filled with lysis buffer containing proteinase K and were incubated at 50 °C for complete digestion. Total DNA was extracted according to the manufacturer’s protocol (Micro AX Tissue Gravity, A&A Biotechnology, Gdynia, Poland).

### 4.4. PCR Conditions

PCR amplification was performed with the primers Al167F 5′-GCG GTC ATA GTT TTC AGG GTT T-3′/Al198R 5′-CTC CGT ATG TGG GAT TTG TAA GC-3′ (608 bp) designed on the basis of the *A. lumbricoides* β-tubulin nucleotide sequence [6]. The 25 µL of PCR mixture contained 12.5 µL DreamTaq Green PCR Master MIX (Thermo Scientific, Waltham, MA, USA), 1 µL of each primer at the concentration of 10 µM, 5.5 µL nuclease-free water and 5 µL of the DNA extracted from each sample. The PCR thermal cycle conditions were as follows: an initial denaturation step took place at 95 °C for 2 min; it was followed by 40 cycles of amplification for 30 s at 94 °C, 45 s at 60 °C and 1 min at 72 °C. Final elongation was performed at 72 °C for 8 min. All reactions were carried out in a Mastercycler Nexus (Eppendorf, Hamburg, Germany). Amplification products were visualized by electrophoresis on 1.5% agarose gel stained with Midori Green DNA Stain (Nippon Genetics Europe GmbH, Düren, Germany). In each PCR run, nuclease-free water as a negative control and positive control samples were used. The positive control for the *A. suum* β-tubulin gene included genomic DNA from a previously positive sample.

### 4.5. PCR-RFLP Analysis

The polymorphisms at codon 167 in the β-tubulin gene were assessed by PCR-RFLP according to Zuccherato et al. [6] with our own modification. The enzyme *RsaI* (Eurix, Gdańsk, Poland) was used for digestion of codon and 167.5 µL of amplified DNA were digested in a Mastercycler Nexus at 37 °C for 60 min. The reaction mixture contained 16.75 µL of nuclease-free water, 0.25 µL of bovine serum albumin (BSA), 2.5 µL of 10× ONE buffer and 0.5 µL of *RsaI* enzyme. PCR-RFLP products were separated by electrophoresis in a 3% agarose gel stained with Midori Green DNA Stain dye. The expected fragment sizes after the digestion of each genotype of codon 167 are shown in Figure 5.

### 4.6. DNA Sequencing and Data Analysis

To confirm genotyping, two randomly chosen PCR products (608 bp) were purified (Clean Up Purification Kit, A&A Biotechnology, Gdynia, Poland) and sequenced in two directions with Al167F/Al198R primers (Macrogen Europe, The Netherlands). Edition of the obtained sequences were conducted using BioEdit software [33]. The BLAST-NCBI program (http://www.ncbi.nlm.nih.gov/BLAST/) was used to identify similarities with deposited β-tubulin gene sequences. The new sequences have been registered in the GenBank database (accession numbers: MT996010, MT996011).

### 4.7. Statistical Analysis

Data were expressed as means ± SD of three independent replicates by one-way ANOVA using GraphPad Prism 8 (GraphPad Software, La Jolla, CA, USA). Tukey’s multiple comparisons test was used to assess differences between means. A *p*-value less than 0.05 (*p* < 0.05) was regarded as statistically significant.

## 5. Conclusions

*S*-methyl-(2-methoxycarbonylamino-benzimidazole-5) thiosulfonate appears to be a promising anti-helminthic agent in the control of ascariasis. The 5 mM drug solution was most effective in inhibiting egg development when treated for 48–72 h. It is expected that the use of the thiosulfonate benzimidazole derivative treatment of pigs infected with ascariasis may contribute to the elimination of parasites and reduce environmental contamination from worm eggs. Such measures may reduce the likelihood of animals being re-infected. For this purpose, comprehensive studies should be carried out on adult roundworms followed by an in vivo study of the drug. The ovicidal effect of the drug should be confirmed by infecting other experimental animals in future studies.
